# Lipoxin A4 improves myocardial ischemia/reperfusion injury through the Notch1-Nrf2 signaling pathway

**DOI:** 10.1515/biol-2025-1200

**Published:** 2025-11-20

**Authors:** Xueyun Yan, Anbang Chen, Yuanqi Gong, Jun Xu, Jinyue Lu, Linjuan Wang, Yunfei Fan, Naijing Gao, Le Zhou, Huaming Cao, Xiaodong Kuang

**Affiliations:** Department of Cardiology, Shi bei Hospital, Shanghai, People’s Republic of China; Department of Pathology, Basic Medical College, Jiangxi Medical College, Nanchang University, Nanchang, Jiangxi, People’s Republic of China; Department of Critical Care Medicine/ICU (Intensive Care Unit), Second Affiliated Hospital of Nanchang University, Nanchang, Jiangxi, People’s Republic of China

**Keywords:** oxidative stress, ischemia/reperfusion, LXA4, microtubules, Notch1-Nrf2.

## Abstract

This study investigates the mechanism by which lipoxin A4 (LXA4) attenuates myocardial ischemia/reperfusion injury (MIRI). Using a rat MIRI model, we evaluated coronary flow, histopathology, cardiac enzymes (cTnI/CK-MB), oxidative stress markers (superoxide dismutase, SOD; malondialdehyde, MDA; glutathione peroxidase, GSH-Px), and protein expression (neurogenic locus notch homolog protein 1 [Notch1], hairy and enhancer of split 1 [Hes1], nuclear factor-erythroid 2-related factor 2 [Nrf2], β-tubulin). Pharmacological inhibitors *N*-[*N*-(3,5-difluorophenacetyl)-l-alanyl]-*S*-phenylglycinet-butylester (DAPT; Notch1) and ML385 (Nrf2) were co-administered with LXA4. Results demonstrated that I/R injury significantly increased microtubule disruption (48.5 ± 6.7% vs control 4.8 ± 0.9%; *p* < 0.01), decreased SOD (42.1 ± 5.3 vs 89.6 ± 8.7 U/mg), and GSH-Px (15.2 ± 2.1 vs 34.8 ± 4.5 U/mg), and elevated MDA (6.9 ± 0.8 vs 2.1 ± 0.3 nmol/mg; *p* < 0.05). Notch1, Hes1, and nuclear Nrf2 decreased by 58, 63, and 52%, respectively. LXA4 treatment reversed these effects (23.6 ± 3.6% microtubule disruption; *p* < 0.05), with DAPT or ML385 abolishing LXA4’s protection. These findings indicate that LXA4 protects against MIRI by preserving microtubule integrity and activating the Notch1/Hes1/Nrf2 pathway, revealing a novel mechanism for oxidative stress suppression in cardiomyocytes.

## Graphical abstract

**Figure:**
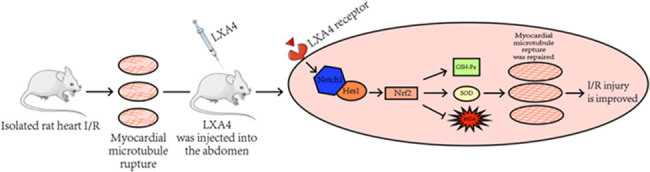

## 1 Introduction

Myocardial ischemia/reperfusion injury (MIRI) represents a critical clinical paradox wherein reperfusion therapy-designed to salvage ischemic myocardium-paradoxically exacerbates tissue damage, leading to arrhythmias, ultrastructural collapse, and cardiomyocyte death [1,2]. This phenomenon arises from a self-perpetuating cascade of inflammatory activation, oxidative stress, and metabolic derangement. Inflammatory cytokines trigger neutrophil infiltration, amplifying injury through protease release and reactive oxygen species (ROS) overproduction [3,4], while mitochondrial dysfunction and calcium overload disrupt energy homeostasis, synergistically activating apoptotic and necroptotic pathways [4,5]. Despite decades of research, current therapies remain palliative, underscoring an urgent need for interventions targeting the root mechanisms of MIRI.

Lipoxin A4 (LXA4), an endogenous pro-resolving mediator, has emerged as a promising candidate for mitigating ischemia/reperfusion (I/R) injury due to its dual capacity to suppress inflammation and enhance tissue repair [6,7,8]. Preclinical studies demonstrate its efficacy in reducing infarct size and improving cardiac function across multiple organ systems [9,10,11]. However, existing research is yet to fully delineate how LXA4 preserves cardiomyocyte ultrastructure, particularly its role in stabilizing microtubule networks essential for mechanical and signaling integrity. Furthermore, while LXA4 is known to modulate oxidative stress, the interplay between its antioxidant effects and redox-sensitive signaling pathways – such as the neurogenic locus notch homolog protein 1 (Notch1)/nuclear factor-erythroid 2-related factor 2 (Nrf2) axis, a central regulator of cellular stress adaptation – remains poorly characterized. These knowledge gaps limit the translation of LXA4’s therapeutic potential into clinically actionable strategies. The Notch1-Nrf2 signaling crosstalk enhances cardiomyocyte viability and antioxidant capacity by activating the Keap1-Nrf2 pathway to suppress mitochondrial ROS generation and improve mitochondrial function, representing a promising therapeutic strategy against myocardial I/R injury [12,13]. However, the specific role and interplay of the Notch1-Nrf2 axis in MIRI, particularly in the context of LXA4 intervention, remain largely unexplored.

This study addresses these limitations by systematically interrogating LXA4’s cardioprotective actions through an integrated framework. First, we explore its ability to maintain microtubule stability, a structural determinant of cardiomyocyte resilience often compromised during I/R injury. Second, we dissect its role in rebalancing oxidative stress by restoring antioxidant enzyme activity (e.g., superoxide dismutase [SOD], glutathione peroxidase [GSH-Px]) and suppressing lipid peroxidation markers (e.g., malondialdehyde [MDA]). Crucially, we unravel the mechanistic link between LXA4 and the Notch1/Nrf2 pathway, demonstrating how this mediator activates Nrf2-driven antioxidant defenses while modulating Notch1-dependent cellular stress responses. By unifying structural, biochemical, and molecular analyses, our work reveals that LXA4 disrupts the vicious cycle of MIRI not through isolated mechanisms, but via coordinated preservation of cellular architecture, redox homeostasis, and stress-adaptive signaling.

Our findings provide the first evidence that LXA4’s cardio protection is mechanistically rooted in its capacity to stabilize microtubule networks and activate the Notch1/Nrf2 axis, offering a dual-target strategy against MIRI. This integrated approach contrasts with conventional therapies that address singular pathological pathways, positioning LXA4 as a multifaceted therapeutic agent. By resolving longstanding uncertainties about its mode of action, this study bridges critical gaps between preclinical promise and clinical application, paving the way for targeted interventions that mitigate reperfusion injury while promoting myocardial recovery.

## 2 Materials and method

### 2.1 Animal models and experimental design

Male Sprague-Dawley rats (8 weeks old, 200–250 g) were obtained from Changsha Tianqin Biotechnology Co., Ltd (China) and maintained under specific pathogen-free conditions at 25 ± 1°C with a 12 h light/dark cycle. Animals were fed irradiated sterile feed and provided *ad libitum* access to water.


**Ethical approval:** The research related to animal use has been complied with all the relevant national regulations and institutional policies for the care and use of animals, and has been approved by the Animal Ethics Committee of Nanchang University (Approval NO. NCULAE-20220624013).

### 2.2 Drug administration and experimental groups

To determine the optimal therapeutic dose of LXA4 (Ann Arbor, MI, USA), a total of 60 rats were randomly assigned to 5 groups (*n* = 12, per group): a control group (sham surgery), I/R group, and three I/R groups receiving intraperitoneal injections of LXA4 at 100, 200, or 400 μg/kg. A subsequent cohort of 72 rats was utilized and expanded to 6 groups (*n* = 12, per group): a subsequent experiment expanded the cohort to 6 groups: control, LXA4 alone (optimal dose), I/R, I/R+LXA4, I/R+LXA4+DAPT (Notch1 inhibitor, 100 μg/kg; Sigma-Aldrich, Germany), and I/R+LXA4+ML385 (Nrf2 inhibitor, 100 μg/kg; Sigma-Aldrich, USA). The doses of *N*-[*N*-(3,5-difluorophenacetyl)-l-alanyl]-*S*-phenylglycinet-butylester (DAPT) (100 μg/kg) and *N*-[4-[2,3-Dihydro-1-(2-methylbenzoyl)-1*H*-indol-5-yl]-5-methyl-2-thiazolyl]-1,3-benzodioxole-5-acetamide (ML385) (100 μg/kg) were selected based on previous studies demonstrating their efficacy in effectively inhibiting the respective target pathways in rodent models of cardiovascular injury without inducing significant off-target toxicity [14,15]. LXA4 and inhibitors were dissolved in dimethyl sulfoxide (DMSO; Solarbio, China) and diluted in saline to ensure a final DMSO concentration <0.1%. All agents were administered intraperitoneally 10–30 min prior to ischemia induction.

### 2.3 Surgical induction of MIRI

MIRI was established as previously described [16,17]. Briefly, rats were anesthetized via intraperitoneal injection of pentobarbital sodium (40 mg/kg) followed by continuous 1.5–2% isoflurane via facemask. Anesthetic depth was confirmed by loss of pedal withdrawal reflex (toe pinch), abolished corneal reflex, and stable respiratory rate (50–70 bpm), with core temperature maintained at 37.0 ± 0.5°C using a heating pad. Following heparinization (300 IU, i.p.), a left thoracotomy was performed to expose the heart. The left anterior descending coronary artery was ligated 2 mm below the left auricle for 30 min to induce ischemia. Hearts were then rapidly excised, immersed in ice-cold Krebs–Henseleit (K-H) buffer, and mounted on a Langendorff perfusion system for retrograde perfusion with oxygenated K-H buffer (95% O_2_/5% CO₂, pH 7.35–7.45) at 37°C and constant pressure (75 mmHg). The *ex vivo* Langendorff model was selected to isolate cardiac-specific mechanisms without confounding systemic factors, allowing precise control over perfusion conditions and drug delivery.

### 2.4 Perfusion protocols and tissue collection

Control hearts underwent continuous perfusion with K-H buffer for 120 min without ischemia. In the I/R group, 30 min of global ischemia preceded 120 min of reperfusion. For intervention groups, LXA4 and/or inhibitors were administered prior to ischemia, followed by identical perfusion protocols. After reperfusion, left ventricular tissue was either immediately frozen in liquid nitrogen for molecular analyses or embedded in optimal cutting temperature (OCT) compound for cryosectioning. After experimental procedures, euthanasia was performed under deep anesthesia by intracardiac injection of potassium chloride (1 M, 0.5 mL/kg) with prior heparinization (300 IU, i.p.), followed by bilateral thoracotomy to confirm death. This protocol adheres to AVMA Guidelines (2020) for rodents >200 g. Transverse sections were stored at −80°C for subsequent immunofluorescence staining of microtubules, while remaining tissue was reserved for biochemical assays and Western blot analysis.

### 2.5 Assessment of coronary blood flow dynamics

Coronary blood flow was quantitatively evaluated before and after I/R by measuring perfusate effluent from the right atrium. Perfusate volume was collected into graduated cylinders over timed intervals, with coronary flow rate calculated as mL/min/g heart weight. Coronary artery blood flow velocity was simultaneously monitored using a calibrated flowmeter, and data were excluded if baseline flow rates fell below 10 mL/min to ensure physiological relevance. Following reperfusion, left ventricular tissue was immediately dissected into two portions: one fixed in 4% paraformaldehyde for histopathological evaluation, and the remaining was snap-frozen in liquid nitrogen for subsequent molecular analyses. All samples were stored at −80°C until processing to preserve biochemical integrity. All assessments were performed in the *ex vivo* setup; no *in vivo* blood flow measurements were conducted. Perfusate effluent was collected over 1 min intervals at baseline, immediately post-ischemia, and at 30 min intervals during reperfusion.

### 2.6 Histopathological evaluation of myocardial tissue

Left ventricular tissues harvested post-reperfusion were fixed in 4% paraformaldehyde for 24 h, followed by sequential dehydration through an ethanol gradient, xylene clearing, and paraffin embedding. Serial Section (4–5 µm thickness) were prepared using a Leica RM2135 microtome (Leica Microsystems, Germany) and stained with hematoxylin and eosin (H&E) for light microscopic analysis (Olympus BX51, Japan). Histopathological alterations, including cardiomyocyte alignment, nuclear integrity, cytoplasmic swelling, and necrosis patterns, were systematically evaluated by blinded observers.

Myocardial injury severity was graded on a standardized 0–4 scale [18,19]: Grade 0 denoted intact tissue architecture with orderly cardiomyocyte arrangement, distinct striations, and absence of pathological changes; Grade 1 indicated focal subendocardial coagulative necrosis without transmural involvement; Grade 2 represented patchy necrosis spanning partial myocardial layers but lacking interlesional connectivity; Grade 3 exhibited confluent necrotic regions with interconnected lesions; Grade 4 corresponded to pan-myocardial necrosis involving the entire ventricular wall, often accompanied by mural thrombus formation. Quantitative analysis incorporated three randomized fields per section to ensure representative sampling.

### 2.7 Enzyme-linked immunosorbent assay (ELISA)

When myocardial injury occurs, troponin I (cTnI) and creatine kinase isoenzyme (CK-MB) are released into the coronary effluent. At the end of the reperfusion period, samples of the coronary effluent were obtained, and the levels of cTnI and CK-MB were evaluated using ELISA kits, following the instructions provided by the manufacturer (Solarbio, Beijing, China). Additionally, some myocardial tissue was homogenized into a 10% (w/v) solution, and the levels of superoxide dismutase (SOD), malondialdehyde (MDA), and glutathione peroxidase (GSH-Px) were evaluated with ELISA kits from the same supplier (Solarbio, Beijing, China).

### 2.8 Western blotting analysis

The tissue from the myocardium was subjected to homogenization through the use of a mechanical homogenizer, and RIPA lysis buffer (Beyotime, Jiangsu, China) was utilized to obtain the total protein content. The quantification of proteins was performed using the BCA Protein Assay Kit (Beyotime, Jiangsu, China). Subsequently, polyacrylamide gel electrophoresis (PAGE) was conducted, and subsequent to this, the samples were transferred to membranes made of polyvinylidene fluoride membranes (Millipore, Eschborn, Germany). A 5% w/v BSA solution was added to phosphate-buffered saline (PBS) containing 0.1% Tween 20 and sealed at ordinary temperatures for 1 h. Primary antibodies, including β-tubulin and β-actin from Santa Cruz Biotechnology (Santa Cruz, CA, USA), and Anti-Notch1, hairy and enhancer of split 1 (Hes1), and Nrf2 antibodies from Abcam (Cambridge, MA, USA), were diluted and added according to the instructions. Suitable secondary antibodies were diluted (1:1,000–1:2,000 v/v) in PBS with 0.1% Tween 20 at ordinary temperatures and incubated for 1 h. Signal detection was performed using the ECL reagent kit (Thermo Scientific Pierce, Rockford, IL, USA).

### 2.9 Assessment of microtubule structural integrity

To evaluate microtubule damage, myocardial tissues were perfused with periodate-lysine-paraformaldehyde fixative immediately following reperfusion, as described in established protocols [20,21]. Left ventricular specimens were excised, rinsed in ice-cold PBS (pH 7.4), and embedded in OCT compound for cryosectioning. Serial frozen sections (10 μm thickness) were incubated overnight at 4°C with a monoclonal anti-β-tubulin primary antibody, followed by a 1 h incubation with fluorescein isothiocyanate-conjugated goat anti-mouse secondary antibody (1:300 dilution; Abcam, Cambridge, MA, USA). After three washes with Tris-buffered saline containing 0.1% Tween-20, sections were mounted with antifade medium and imaged under a fluorescence microscope (BX51; Olympus, Tokyo, Japan) using standardized exposure parameters to ensure comparability across samples.

### 2.10 Quantitative analysis of microtubule disruption

Microtubule structural integrity was quantified using a validated scoring protocol¹⁸. For each specimen, 20–40 high-power fields (400× magnification) were randomly selected from 10 serial sections, with a focus on regions exhibiting diminished or absent β-tubulin fluorescence. Cross-sectional orientations were systematically excluded to standardize planar analysis. Digital images were spatially calibrated to a uniform scale (18 cm × 12 cm), and individual cardiomyocytes spaced at least 1 cm apart were demarcated as regions of interest. Areas containing continuous microtubule lattices were classified as intact zones, while regions with fragmented or dissolved microtubules were designated disrupted zones. The microtubule disruption index was calculated as the percentage of the total disrupted zone area relative to the combined area of intact and disrupted zones within each analyzed field [22].

### 2.11 Statistical analysis

The data were expressed as mean value ± standard deviation (SD). The analysis of differences between pairs of groups was conducted using a two-tailed *t*-test as proposed by Student, whereas comparisons across multiple groups were evaluated using one-way ANOVA, followed by Tukey’s post-hoc analysis. Analyses were performed in GraphPad Prism 8.0 (GraphPad Software, San Diego, CA, USA). All histograms were assessed using this methodology. The statistical examination was conducted via Stata 11.0 (SAS Inc, Cary, NC, USA). A significance level of *P* < 0.05 was considered indicative of statistical difference.

## 3 Results

### 3.1 LXA4 attenuates MIRI in *ex vivo* rat hearts

Histopathological analysis revealed significant myocardial damage in the I/R group, with pathology scores markedly elevated compared to controls (Table 1). LXA4 administration (100–400 μg/kg) dose-dependently reduced myocardial injury severity, achieving maximal protection at 200 μg/kg (##*p* < 0.01 vs I/R group). Notably, no additional benefit was observed at the 400 μg/kg dose, suggesting a ceiling effect in therapeutic efficacy (*p* > 0.05, 200 vs 400 μg/kg).

**Table 1:** Effect of LXA4 on pathological changes in I/R myocardium

Group	Dose (µg/kg)	Pathological grades					
		0	I	II	III	IV	*P* value
Control	—	7	1	0	0	0	
I/R	—	0	0	2	2	4	**
I/R+LXA4	100	2	1	4	1	0	#
	200	1	5	2	0	0	##
	400	1	6	1	0	0	##

** vs control group (*p* < 0.01); # vs I/R group (*p* < 0.05); ## vs I/R group (*p* < 0.01).

**Figure 1:**
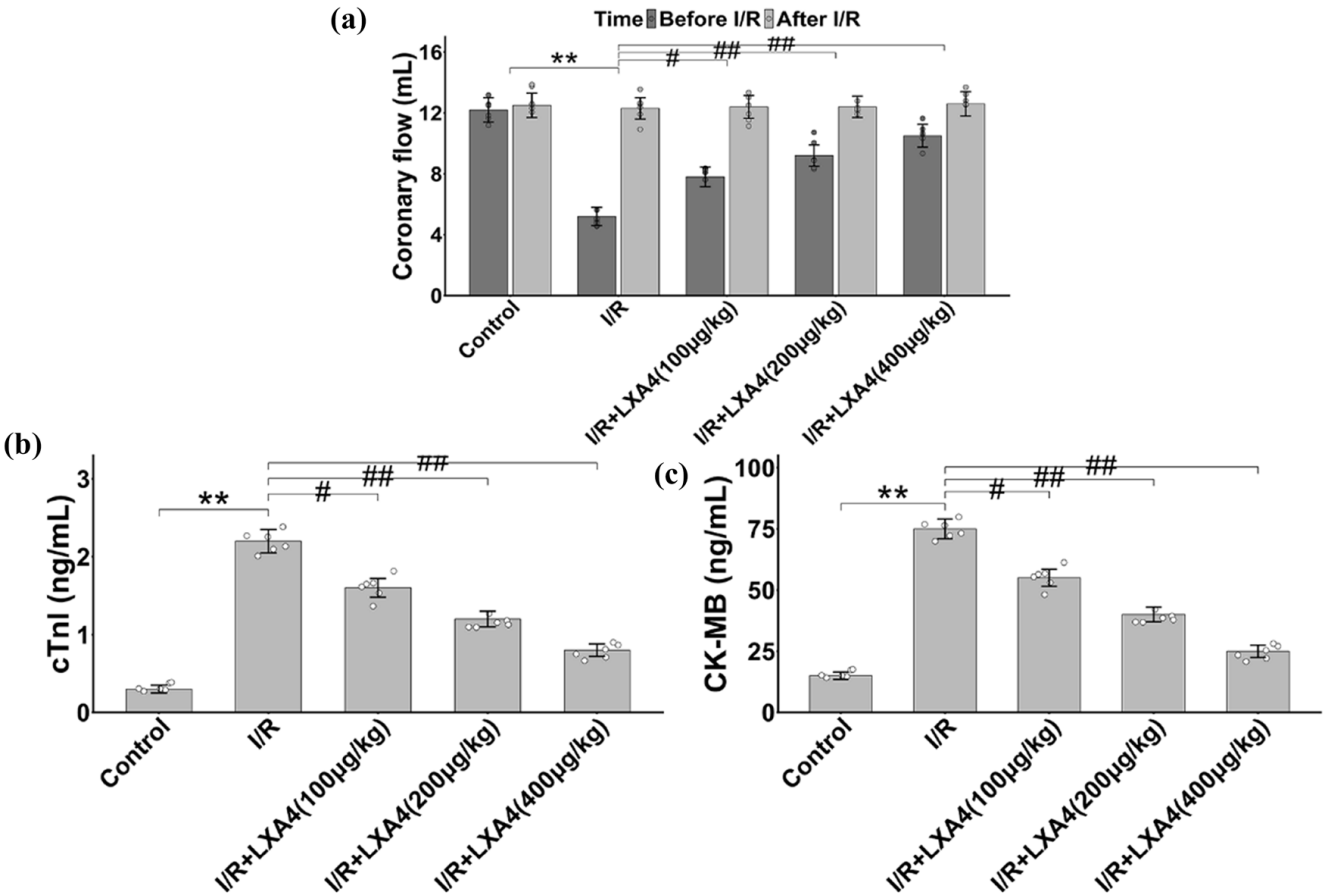
The application of LXA4 facilitates the mitigation of injury caused by I/R in isolated rat cardiac tissues. (a) Effect of LXA4 on coronary blood flow during I/R in isolated rat cardiac. (b) and (c) Effects of LXA4 on the content of cTnI and CK-MB after I/R in rats. ** vs control group (*p* < 0.01); # vs I/R group (*p* < 0.05); ## vs I/R group (*p* < 0.01).

Hemodynamic assessments demonstrated preserved coronary blood flow (CF) in LXA4-treated hearts post-I/R. While baseline CF remained comparable across groups, I/R injury induced a sharp decline in CF (Figure 1a). LXA4 treatment (100–400 μg/kg) restored CF to near-physiological levels, with 200 μg/kg yielding optimal recovery (##*p* < 0.05 vs I/R group). Biochemical analysis further corroborated these findings: serum cTnI and CK-MB levels, elevated 3.2-fold and 2.8-fold in the I/R group, respectively, were normalized by LXA4 in a dose-dependent manner (Figure 1b and c).

Collectively, these data confirm successful induction of the I/R model and establish 200 μg/kg as the minimally effective dose for LXA4-mediated cardioprotection. This dose was selected for subsequent mechanistic studies to avoid potential off-target effects associated with higher concentrations.

### 3.2 LXA4 can restore microtubule expression and protect microtubule structure in I/R injury

**Figure 2:**
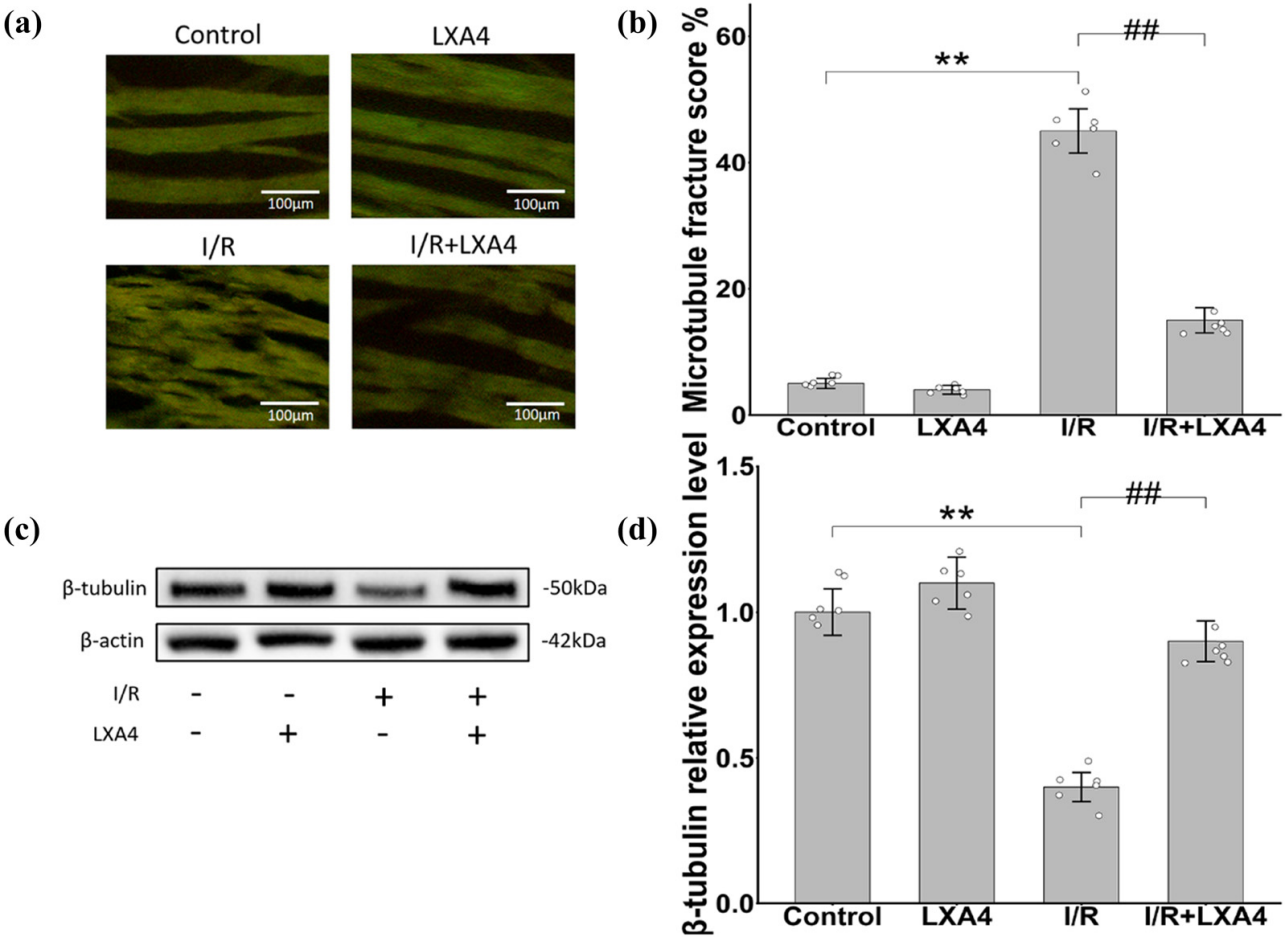
LXA4 alleviates microtubule damage caused by myocardial ischemia in rats. (a) The microtubule rupture levels of Control group, LXA4 group, I/R group, and I/R+LXA4 group were detected by immunofluorescence; (b) Microtubule rupture score histogram. (c) and (d) The relative expression level of β-tubulin. ** vs control group (*p* < 0.01); ## vs I/R group (*p* < 0.01).

Immunofluorescence analysis revealed a noteworthy contrast: in comparison to the control group (4.8 ± 0.9%), the microtubule rupture score in the I/R group exhibited a substantial increase to 48.5 ± 6.7%. Following treatment with LXA4 (200 μg/kg), the microtubule rupture score showed a marked reduction to 23.6 ± 3.6%. It is worth noting that no significant differences were observed between LXA4 alone group (200 μg/kg) (4.7 ± 1.1%) and the control group (Figure 2a and b). Complementing these results, β-tubulin expression was significantly reduced in the I/R group compared with the normal group, but treatment with LXA4 (200 μg/kg) markedly restored β-tubulin levels (Figure 2c and d).

### 3.3 LXA4 can regulate the oxidative stress in I/R damage

**Figure 3:**
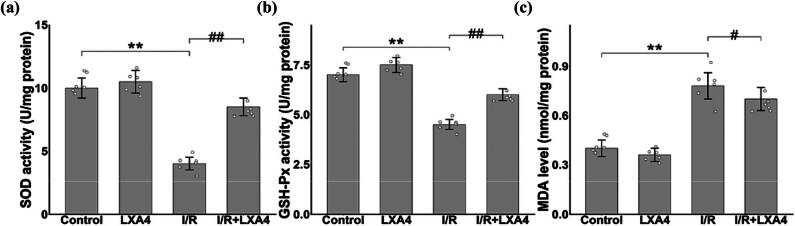
The application of LXA4 mitigates the harmful effects of oxidative stress induced by I/R. (a) Changes in SOD activity after I/R; (b) Changes in GSH-Px activity after I/R; (c) Changes in MDA content after I/R.** vs control group (*p* < 0.01); # vs I/R group (*p* < 0.05); ## vs I/R group (*p* < 0.01).

I/R damage is frequently linked to oxidative stress, necessitating the assessment of key oxidative stress indexes such as SOD, GSH-Px, and MDA. The findings revealed notable alterations compared to the control group: in the I/R group, the antioxidant markers SOD (Figure 3a) and GSH-Px (Figure 3b) exhibited a significant decrease, whereas the oxidant indicator MDA (Figure 3c) demonstrated a marked increase. Following LXA4 treatment for I/R injury, there was a substantial increase in SOD and GSH-Px levels, accompanied by a significant decrease in MDA levels. These results strongly imply that oxidative stress plays a crucial role in I/R damage, and the administration of LXA4 can effectively ameliorate the I/R condition by reducing oxidative stress.

### 3.4 LXA4 enhanced the recovery from I/R damage through the Notch1-Nrf2 signaling pathway

**Figure 4:**
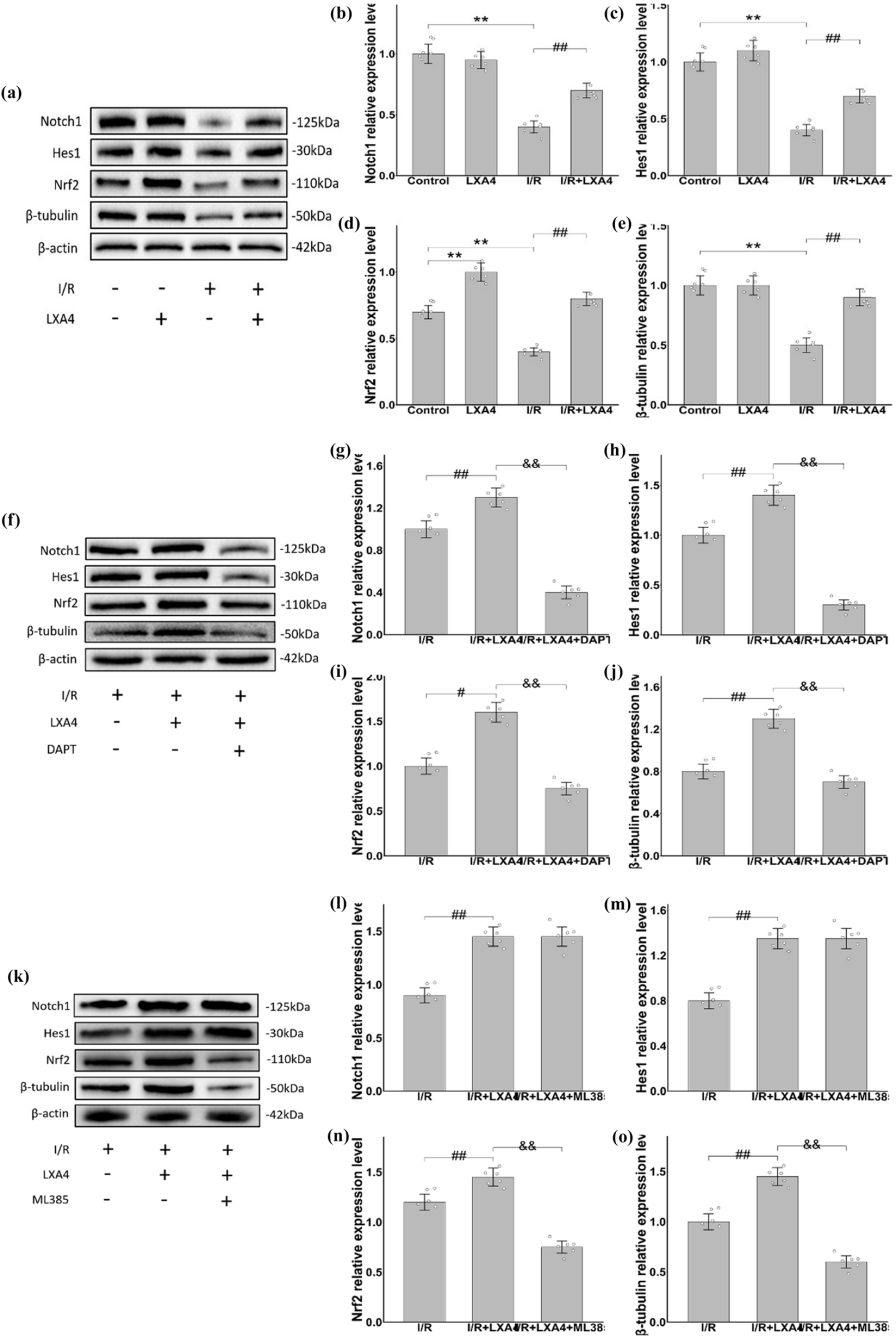

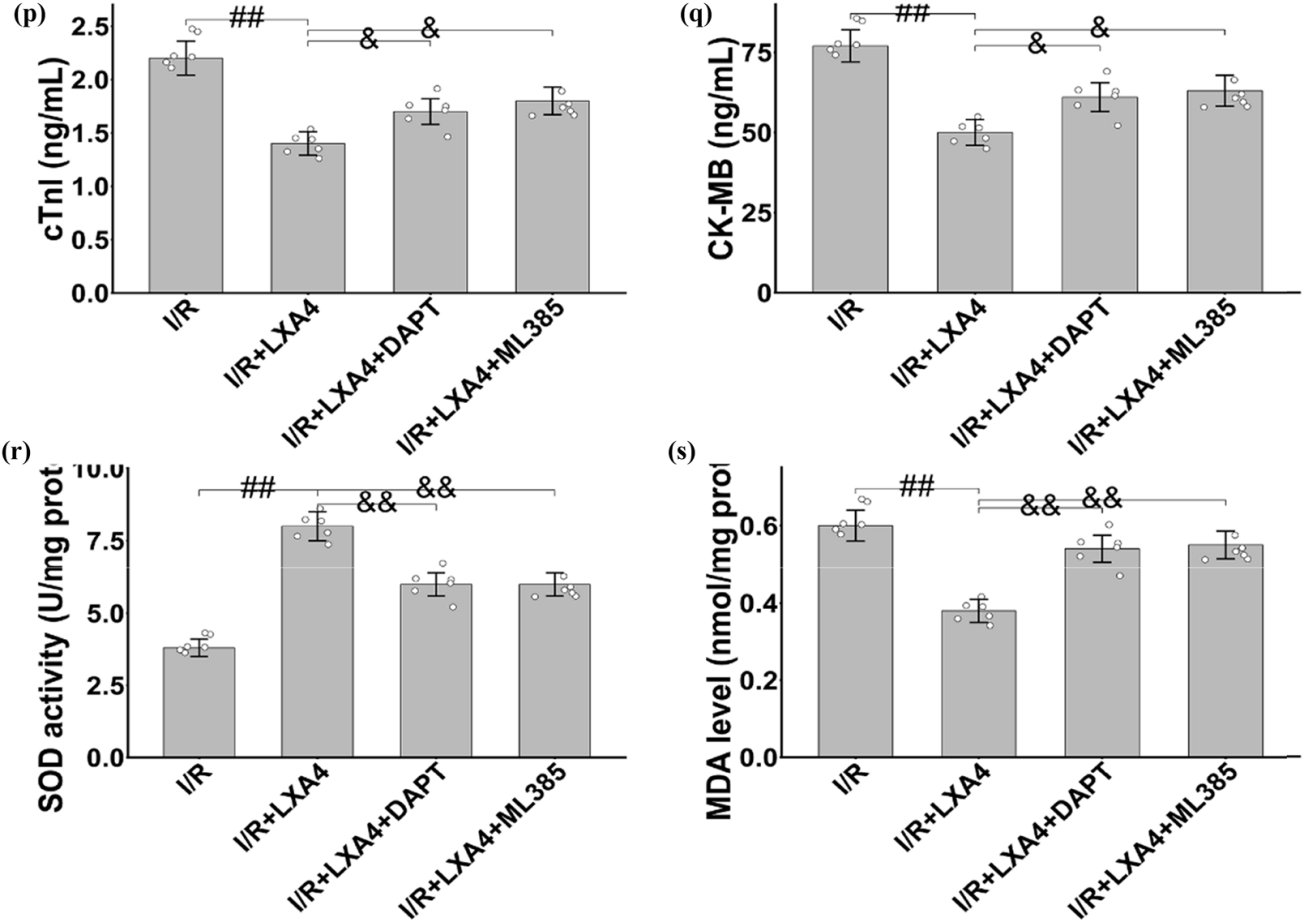
LXA4 attenuates MIRI via the Notch1-Nrf2 signaling pathway in isolated rat hearts. (a) Representative Western blot images demonstrating protein expression levels of Notch1, Hes1, Nrf2, and β-tubulin across experimental groups. (b)–(e) Densitometric quantification of relative protein expression levels of Notch1 (b), Hes1 (c), Nrf2 (d), and β-tubulin (e). (f) Western blot analysis of Notch1, Hes1, Nrf2, and β-tubulin expression after DAPT (Notch1 inhibitor) administration. (g)–(j) Quantitative analysis of Notch1 (g), Hes1 (h), Nrf2 (i), and β-tubulin (j) expression after DAPT administration. (k) Western blot profiles showing protein expression of Notch1, Hes1, Nrf2, and β-tubulin after ML385 (Nrf2 inhibitor) administration. (l)–(o) Densitometric evaluation of Notch1 (l), Hes1 (m), Nrf2 (n), and β-tubulin (o) levels after ML385 administration. (p)–(s) Biochemical markers of myocardial injury and oxidative stress: (p) cTnI, (q) CK-MB, (r) SOD activity, and (s) MDA content. Data are presented as mean value ± SD (*n* = 6). ** vs control group (*p* < 0.01); # vs I/R group (*p* < 0.05); ## vs I/R group (*p* < 0.01); & vs I/R+LXA4 group (*p* < 0.05); && vs I/R+LXA4 group (*p* < 0.01).

The results show that the expression level of Notch1, Hes1, Nrf2, and β-tubulin in the I/R group were significantly reduced compared to the control group. However, after LXA4 treatment, the protein levels of Notch1, Hes1, Nrf2, and β-tubulin exhibited a significant upregulation (Figure 4a–e). This implies that LXA4 enhances the expression of β-tubulin, closely associated with the Notch1/Nrf2 signaling pathway.

Subsequently, we employed DAPT (a Notch1 inhibitor) and ML385 (an Nrf2 inhibitor). In comparison to the I/R+LXA4 group, the upregulation of Notch1, Hes1, Nrf2, and β-tubulin was significantly inhibited after the addition of DAPT (Figure 4f–j). Additionally, the expressions of Nrf2 and β-tubulin were significantly suppressed following the addition of ML385, while the upregulation of Notch1 and Hes1 remained unchanged compared to the I/R+LXA4 group (Figure 4k–o). This indicates that Nrf2 is a downstream protein of Notch1.

ELISA results demonstrated that upon the addition of inhibitors, in comparison to the I/R+LXA4 group, the levels of cTnI (Figure 4p), CK-MB (Figure 4q), and MDA (Figure 4s) significantly increased, while the content of SOD (Figure 4r) decreased significantly. These findings suggest that LXA4 regulates oxidative stress and microtubule expression, thereby alleviating I/R damage via the Notch1-Nrf2 signaling pathway.

## 4 Discussion

LXA4 has demonstrated potential in treating various conditions such as pulmonary bronchial dysplasia, psoriasis, subarachnoid hemorrhage, testicular torsion injury, sepsis-related liver function impairment, and MIRI [23,24]. Despite its efficacy across these ailments, the precise mechanism underlying LXA4’s effectiveness in the context of I/R injury are not yet fully understood.

Previous investigations have shed light on the complex aspects of I/R injury, implicating pathways like NF-κB, Keap1-Nrf2, HIF-1α/BNIP3, and mitochondrial dysfunction [25,26,27,28]. Furthermore, the intricate microtubule ultrastructure emerges as a critical factor in I/R damage. Myocardial ischemia and hypoxia instigate changes in ion channels, cellular morphology, structural impairment, disarray in microtubule organization, and even disruption of the cytoskeletal framework [29,30]. Our study observed a decline in antioxidant markers (SOD and GSH-Px) alongside an increase in the oxidation index MDA and the microtubule rupture score within the I/R group. Conversely, administration of LXA4 mitigated oxidative stress markers and attenuated microtubule rupture, along with regulating β-tubulin expression. These findings suggest that LXA4 shields against I/R injury by modulating oxidative stress. Notably, it also exhibits a regulatory effect on microtubules, possibly intertwined with its impact on oxidative stress pathways.

Recent investigations have highlighted the signaling pathways of Notch1 and Nrf2 as novel targets for myocardial protection [13]. Nrf2 plays a crucial role in mediating cellular responses to oxidative stress, effectively regulating the expression of key components involved in the antioxidant defense system, such as glutathione and thioredoxin [31,32]. Additionally, Nrf2 actively engages in the clearance of ROS, detoxification of both endogenous and exogenous substances, and the regeneration of NADPH [33,34,35]. In the presence of cellular oxidative stress, Nrf2 becomes activated, leading to the expression of downstream target genes such as HO-1, NQO-1, and GPX4. This activation, in turn, facilitates the elimination of accumulated ROS, consequently mitigating oxidative damage.

Additionally, Nrf2 is intricately linked with various signaling pathways, notably the Notch1 signaling pathway [13]. Upon ligand binding, Notch1 undergoes prompt cleavage and translocates into the nucleus, initiating the transcription of downstream target genes, including Hes1 and Nrf2. This process executes the antioxidant functions of Notch1 [36]. Notch1 target genes, Hes1 and Hes3, can elevate Nrf2 expression, reducing oxidative stress by limiting ROS formation [37]. Interestingly, Nrf2 binds directly to a functional antioxidant response element on the Notch1 promoter, thereby promoting Notch1 expression [38]. These findings underscore the coordinated interplay between the Notch1 and Nrf2 signal pathway, in maintaining cellular oxidative homeostasis. In our study, we employed the Notch1 inhibitor DAPT and Nrf2 inhibitor ML385 to regulate the Notch1 and Nrf2 signal pathway, exploring the intricacies of Notch1-Nrf2 interactions in myocardial protection [38,39]. Our study identified Notch1 as an upstream regulator of Nrf2, consistent with existing literature [40,41]. Furthermore, our findings demonstrate that LXA4 can modulate oxidative stress via the Notch1-Nrf2 signaling pathway. This regulation influences microtubule structure, ultimately mitigating I/R injury.

While our results demonstrate that the administration of DAPT and ML385 effectively attenuated the pathological processes, aligning with the inhibition of the Notch1 and Nrf2 pathways, respectively, it is important to consider the limitations of pharmacological inhibitors. DAPT, as a γ-secretase inhibitor, can potentially affect the cleavage of other substrates beyond Notch1, such as other Notch receptors (Notch2-4) or amyloid precursor protein. Similarly, while ML385 is a selective Nrf2 antagonist, its effects on interconnected signaling networks cannot be entirely ruled out. Therefore, although our pharmacological data strongly suggest the involvement of Notch1 and Nrf2, the observed effects could potentially involve off-target mechanisms. This underscores that inhibitor studies alone are not definitive proof of pathway order or exclusive involvement. To establish a more direct causal relationship, future studies employing genetic approaches, such as cell-specific knockout or knockdown of Notch1 and Nrf2, are essential to corroborate these findings. Despite this limitation, our data provide a solid pharmacological foundation for the functional roles of these pathways in our model and highlight their potential as therapeutic targets.

Recent advances in LXA4 research have further elucidated its role in modulating oxidative stress and inflammation through the Nrf2 pathway. LXA4 has been shown to restore oxidative stress-induced vascular endothelial cell injury by activating the Nrf2-HO-1 axis, thereby offering a promising strategy for managing venous thromboembolism and its complications [42]. Furthermore, LXA4 has been reported to protect against renal ischemia/reperfusion injury by promoting the IRG1/Nrf2 and IRAK-M-TRAF6 signaling pathways, providing a novel strategy for preventing and treating acute kidney injury [43].

In summary, the Nrf2 signaling pathway, in conjunction with Notch1 and LXA4, plays a pivotal role in the regulation of oxidative stress across various disease models. The intricate crosstalk between these pathways offers valuable insights into potential therapeutic targets for managing oxidative stress-related diseases. The continued exploration of these interactions holds promise for the development of novel therapeutic strategies aimed at mitigating the deleterious effects of oxidative stress in human health.

These findings suggest that LXA4, serving as a potential protective agent against I/R injury, may mitigate damage to the microtubule structure through the Notch1-Nrf2 signaling pathway. However, further investigation using transgenic cell or mouse models is necessary to fully be aware of the impact of the Notch1-Nrf2 pathway on the protective effects of LXA4 against MIRI. Future investigations will aim to explore the precise mechanism of Notch1-Nrf2 on LXA4-mediated protection against MIRI in cell models.
